# Supplementary material for: MetaRibo-Seq measures translation in microbiomes
Source: Nat Commun. 2020 Jun 29;11:3268. doi: 10.1038/s41467-020-17081-z (PMC7324362; doi:10.1038/s41467-020-17081-z)
Supplement: Supplementary file 10 — Supplementary Data 7 [file 41467_2020_17081_MOESM10_ESM.zip › File2/Confidence_High_Taxonomy/8251_out.krona.html]

Javascript must be enabled to view this page.

members
magnitude
magnitudeUnassigned
count
unassigned
taxon
rank

8251\_out

3

2
superkingdom
3

phylum
3
976

117743
class
3

200644
order
3

49546
family
3

3
genus
1016


SRS063485\_contig\_number\_15067
species
1
712213

species

SRS018826\_contig\_number\_4674
1
45242

1
species

SRS017814\_contig\_number\_contig-100\_6165.32620
1705617
